# A new HDAC inhibitor cinnamoylphenazine shows antitumor activity in association with intensive macropinocytosis

**DOI:** 10.18632/oncotarget.14714

**Published:** 2017-01-18

**Authors:** Bing-yan Zhu, Bo-yang Shang, Yue Du, Yi Li, Liang Li, Xian-dong Xu, Yong-su Zhen

**Affiliations:** ^1^ Institute of Medicinal Biotechnology, Chinese Academy of Medical Sciences and Peking Union Medical College, Beijing 100050, China

**Keywords:** macropinocytosis, HDAC inhibitors, trans-cinnamic acid, neutral red

## Abstract

Previous studies have shown that intensive macropinocytosis occurs in cancer cells and neutral red (NR) is noted for its capability to enter into the cell massively through a process mimetic to macropinocytosis. In addition, trans-cinnamic acid (tCA) has been found to be an inhibitor of histone deacetylase (HDAC). In the present study, cinnamoylphenazine (CA-PZ) that consists of NR and tCA moieties was synthesized and evaluated. As shown, CA-PZ massively entered into colon carcinoma HT-29 cells and pancreatic carcinoma MIA PaCa-2 cells and this entry was blocked by 5-(N-ethyl-N-isopropyl) amiloride (EIPA, an inhibitor of macropinocytosis), indicating a macropinocytosis-mediated uptake. Furthermore, CA-PZ markedly increased the protein expression levels of acetyl-H3, acetyl-H4 and p21 in HT-29 cells and MIA PaCa-2 cells. CA-PZ significantly inhibited the growth of colon carcinoma HT-29 and pancreatic carcinoma MIA PaCa-2 xenografts. By *in vivo* imaging, CA-PZ displayed prominent accumulation in the tumor xenografts. The study indicates that the newly synthesized CA-PZ acts as an HDAC inhibitor in association with intensive macropinocytosis-mediated intracellular delivery in cancer cells. The use of neutral red for preparation of chimeric molecules with the attribute of macropinocytosis-mediated intracellular delivery might open an alternative way for development of HDAC inhibitors.

## INTRODUCTION

Recent studies have demonstrated that macropinocytosis is significantly activated in a variety of cancer cells. Macropinocytosis is exploited by a range of pathogens for cellular invasion and avoidance of immune surveillance [1]. Among known endocytic routes, macropinocytosis provides a unique pathway for intracellular entry with a form of bulk uptake and can therefore efficiently and rapidly internalize the carried substances [2]. Notably, macropinocytosis is markedly enhanced in K-ras-transformed pancreatic carcinoma cells; apparently, it may represent an important route of tumor nutrient uptake [3]. Besides playing important roles in a range of physiological processes, macropinocytosis is highly relevant to cell migration and tumor metastasis [4, 5]. Macropinocytosis could be induced by the stimulation of epidermal growth factor receptor (EGFR), leading to the enhancement of cellular uptake [6]. In addition, macropinocytosis may be stimulated by a nucleolin-dependent mechanism [7].

Macropinocytosis may be relevant to the expression of histone deacetylase (HDAC). As shown, membrane ruffle formation, macropinocytosis, and cell migration are all impaired in HDAC 6-deficient cells; conversely, elevated HDAC6 levels promote membrane ruffle formation associated with the increase of macropinocytosis and cell motility [8]. HDAC plays an important role in epigenetic mechanisms. The disturbance in histone acetylation or deacetylation may cause alterations in the transcriptional regulation of tumor suppressor genes or oncogenes. Therefore, the changes in histone acetylation may influence gene transcription [9]. Studies have found that cancer is linked to histone hypoacetylation which arises from HDAC overexpression; and the anti-cancer effects of HDAC inhibitors have been attributed to the restoration of histone acetylation balance [10–12]. As known, HDACs are a vast family of enzymes and play crucial roles in numerous biological processes and their expression levels may vary among different cancer cells. HDAC1 is overexpressed in prostate and gastric cancers, where it signalizes poor prognosis [13–15]. High levels of HDAC2 have been found in colorectal, cervical and gastric cancers [16, 17]. HDAC3 is overexpressed in gastric, prostate and colorectal cancer [18]; and high expression level of HDAC1 and HDAC2 correlates with decreased patient survival in colorectal carcinomas [19, 20]. Various HDACs display different intracellular localization. HDAC1, 2, 3, 8 and 11 are mainly found in the nucleus, while HDAC6 and 10 are primarily found in the cytoplasm. However, HDAC4, 5, 7 and 9 can be found both in the nucleus and the cytoplasm. Studies have indicated that HDACs are potential novel therapeutic targets. A number of HDAC inhibitors have reached clinical trials and several being approved for cancer treatment [21–25]. The most extensively used biomarkers for the investigation of HDAC inhibitors were H3 and H4 [26]. In addition, many HDAC inhibitors were found to increase the levels of p21 in a concentration dependent manner [27]. In tumor cells, HDAC inhibitor-induced activation of p21 is controlled by a cross-talk of reversible phosphorylation and acetylation signals [28]. HDAC inhibition may disturb the balance between pro- and anti-apoptotic proteins, causing tumor cell death [29–31].

Cinnamic acid has been found to display a broad spectrum of biological effects including antioxidant, anti-inflammatory and anticancer activities [32–34]. Our previous study has demonstrated that trans-cinnamic acid (tCA) is active as an HDAC inhibitor and it is effective against colon carcinoma xenograft [35]. In addition, neutral red (NR), 3-amino-7-dimethylamino-2-methylphenazine, is a well-known compound that enters into the cell massively by the process of macropinocytosis. Considering the facts that HDAC collectively is an intracellular localized target and tCA can act as an HDAC inhibitor and that neutral red can enter the cell by intensive macropinocytosis, it is of interest to design and prepare a new HDAC inhibitor endowed with the capacity of both intensive macropinocytosis and active HDAC inhibition. The present study is set to synthesize a new compound cinnamoylphenazine (CA-PZ) that integrates NR and tCA, to assess the effect of CA-PZ on HDAC in cancer cells and its antitumor efficacy; moreover, to investigate its molecular mechanism of action.

## RESULTS

### Preparation and purification of CA-PZ

**Figure 1:**
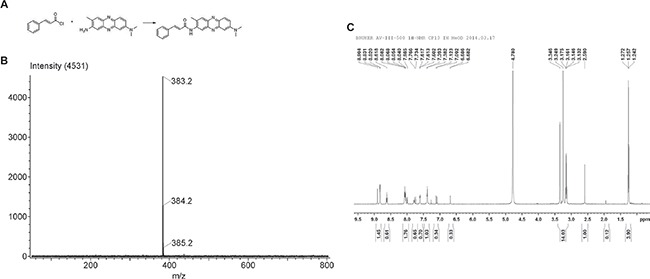
The synthetic compound cinnamoylphenazine (CA-PZ) (**A**) Scheme for the condensation reaction. Reagents and conditions: pyridine, DMF, 60°C, 72 h. (**B**) ESI MS of CA-PZ: 383.2, 384.2, and 385.2, respectively. The identified molecular ion peak (m/z) of CA-PZ is 383.2 [M+H] ^+^ and the molecular weight is 382.1794. The molecular formula is C_24_H_22_N_4_O. (**C**) ^1^H NMR (300 MHz) of CA-PZ. The identified molecular ion peak (m/z) of CA-PZ is 383.2 [M+H] ^+^ and the molecular weight is 382.1794. The molecular formula is C_24_H_22_N_4_O.

The synthetic compound CA-PZ that integrates tCA and NR was prepared by condensation reaction. The electrospray ionization mass spectrometry (MS-ESI) and ^1^H NMR (300 MHz) detection confirmed the conjugation. By high performance liquid chromatography, the purity of the synthetic sample was 95.8%. The molecular formula for CA-PZ is C_24_H_22_N_4_O and molecular weight is 382.1794. MS-ESI showed that the main peak was 383.2. ^1^H NMR (300 MHz) showed the proper number of hydrogen ion in each group (Figure 1).

### Macropinocytosis-mediated uptake of CA-PZ in carcinoma cells

**Figure 2:**
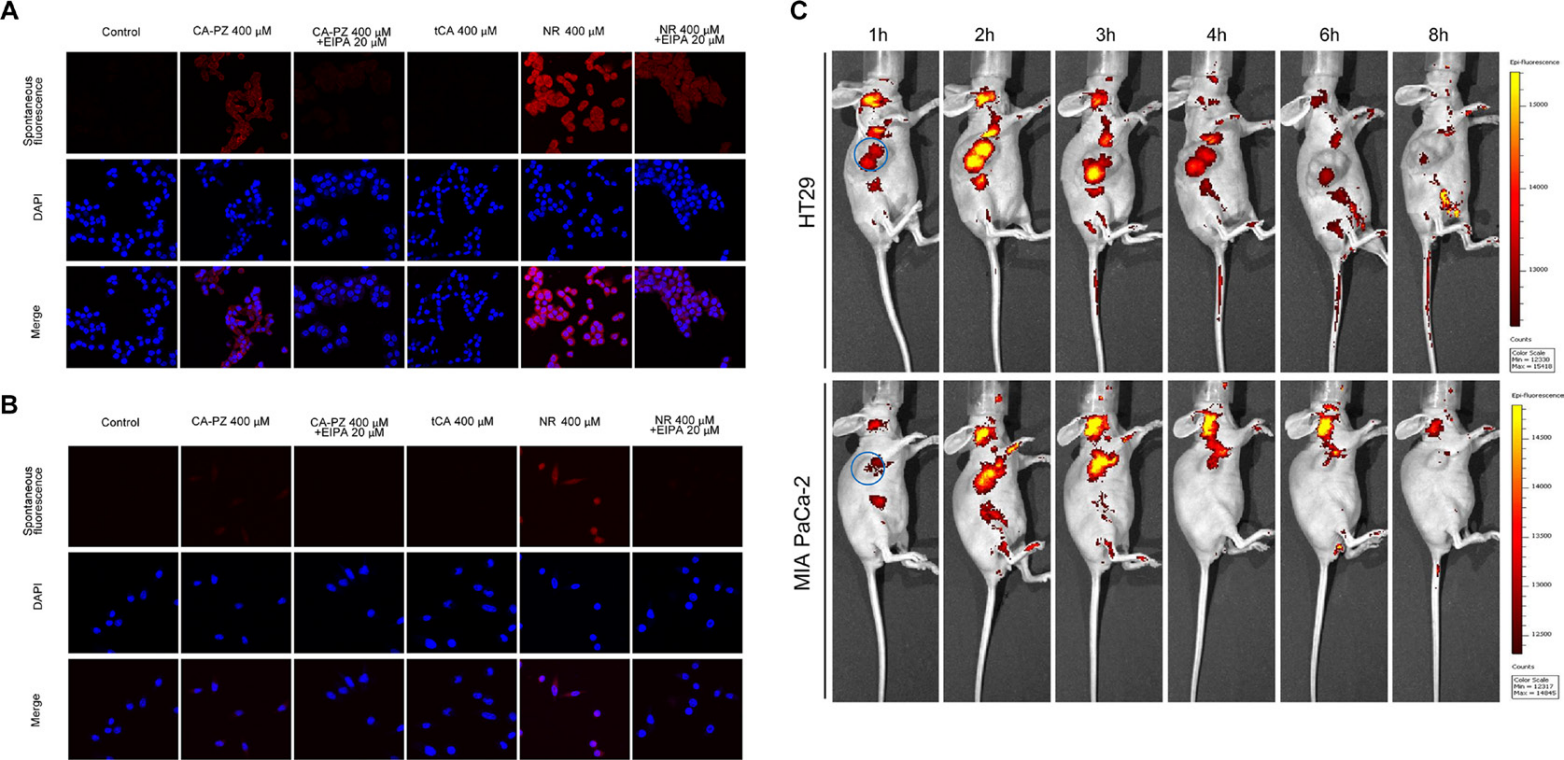
Macropinocytosis assay in colon carcinoma HT29 cells and pancreatic carcinoma MIA PaCa-2 cells (**A**) Images of HT29 cells taken by confocal microscopy. As shown, the CA-PZ treated HT29 cells that displayed appreciably high level of macropinosome formation as seen by red fluorescence were similar to NR-treated cells, whereas the control group and tCA group show almost no fluorescence. In addition, the uptake was inhibited by 5-(N-ethyl-N-isopropyl) amiloride (EIPA), a relative specific inhibitor of macropinocytosis. (**B**) Images of MIA PaCa-2 cells taken by fluorescence microscopy. The appearance of MIA PaCa-2 cells was similar to HT29 cells. (**C**) *In vivo* optical imaging in HT29 and MIA PaCa-2 xenograft-bearing athymic mice after intravenous injection of CA-PZ. Representative *in vivo* fluorescence images at appointed times after tail vein injection of 1.5 mmol/kg CA-PZ. The blue circled area indicates tumor location. Color scale represents photons/s/cm^2^/steradian.

As shown by confocal microscopy, there occurred a massive uptake of CA-PZ both in HT29 cells and MIA PaCa-2 cells. In HT29 cells, the CA-PZ treated cells displayed appreciably high level of macropinosome formation as seen by red fluorescence; apparently, the intensity was comparable to that in NR-treated cells. However, the tCA-treated cells and the controls almost showed no fluorescence. In addition, the uptake of CA-PZ and NR was inhibited by 5-(N-ethyl-N-isopropyl) amiloride (EIPA), an inhibitor of macropinosome formation (Figure 2A). Similar results were obtained in the case of MIA PaCa-2 cells; as shown, the inhibition by EIPA was much more obvious (Figure 2B).

### Inhibition of cancer cell proliferation by CA-PZ

**Figure 3:**
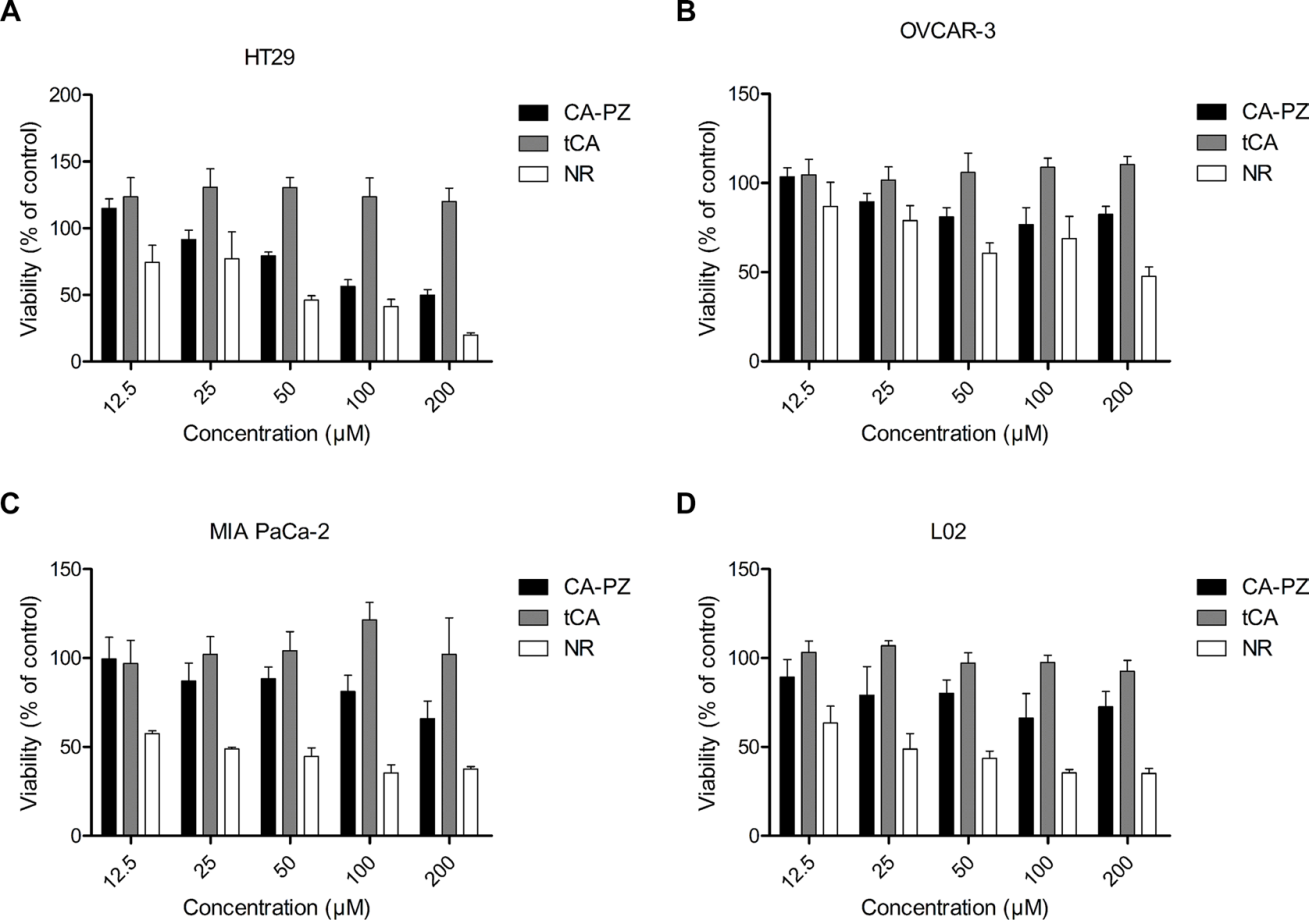
Cell viability for different cancer cell lines and the non-cancerous cell line Cells were seeded at a density of (3–5) × 10^3^/well in 96-well plates and treated with various concentrations of CA-PZ, tCA and NR for 72 h, respectively. (**A**) HT29 colon carcinoma cells. (**B**) OVCAR-3 ovarian carcinoma cells. (**C**) MIA PaCa-2 pancreatic carcinoma cells. (**D**) The non-cancerous L02 hepatic cells. The results are means of triplicates from three separate experiments. As shown, CA-PZ inhibited cancer cell proliferation. HT29 cells were relatively more sensitive to CA-PZ; in addition, CA-PZ was more potent than tCA against various cancer lines. By contrast, the non-cancerous L02 hepatic cells were apparently insensitive to CA-PZ.

Determined by MTT assay, CA-PZ in a concentration-dependent manner inhibited the proliferation of various cancer cell lines, including HT29, MIA PaCa-2, OVCAR3, and L02 (Figure 3). As shown, HT-29 cells were relatively more sensitive to CP-PZ. The IC50 values of CA-PZ for HT29, OVCAR-3, and MIA PaCa-2 cancer cells were 121.6 ± 9.6 μM, 186.9 ± 3.0 μM, and 292.9 ± 10.7 μM, respectively. The IC50 values of tCA for all the tested cancer cell lines were over 500 μM. In comparison, the IC50 value of CA-PZ for L02 cells was 1152.8 ± 31.3 μM, indicating the non-cancerous cells were rather insensitive to CA-PZ (Figure 3D).

### Increase of histone acetylation in cancer cells

**Figure 4:**
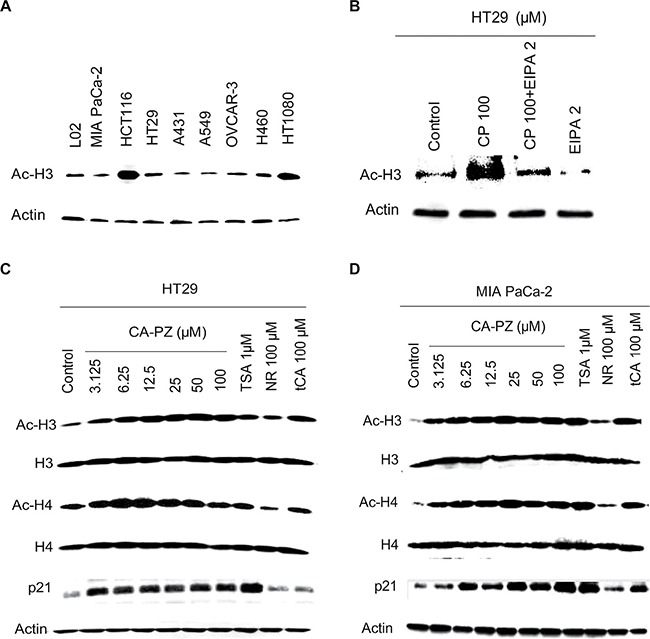
Western blot analysis on the expression of HDAC-related proteins (**A**) Expression levels of acetylated histones H3 in various cancer cell lines and the hepatic L02 cells. (**B**) and (**C**) Expression levels of acetylated histones H3 and H4, and p21 proteins in HT-29 cells treated with various concentrations of CA-PZ (3.125 μM, 6.25 μM, 12.5 μM, 25 μM, 50 μM, and 100 μM) for 12 h. As shown, the expression levels of Ac-H3, Ac-H4, and p21 proteins were increased. Notably, tCA and the known HDAC inhibitor TSA (1 μM) caused similar changes. However, NR (100 μM) caused less change. (**D**) Expression levels of acetylated histones H3 and H4, and p21 proteins in MIA PaCa-2 cells treated with various concentrations of CA-PZ. Similar changes as those in HT-29 cells were found.

To determine whether CA-PZ treatment could increase the acetylation of histone proteins in HT29 cells and MIA PaCa-2 cells, Western blot analysis of the extracted proteins from various cell lines, especially HT29 and MIA PaCa-2 cells, was performed (Figure 4). As shown, the expression levels of acetylated histones H3 were highly varied in different cell lines (Figure 4A). Notably, CA-PZ induced histone acetylation dose-dependently in HT29 cells, resulted in the accumulation of Ac-H3, Ac-H4, and p21 proteins. tCA and the well-known HDAC inhibitor trichostatin A (TSA) induced similar effects (Figure 4B, 4C). Similarly, CA-PZ induced histone acetylation dose-dependently in MIA PaCa-2 cells, resulting in the accumulation of Ac-H3, Ac-H4, and p21 proteins. tCA and TSA induced similar effects as those found in HT-29 cells (Figure 4D).

### Induction of apoptosis by CA-PZ

**Figure 5:**
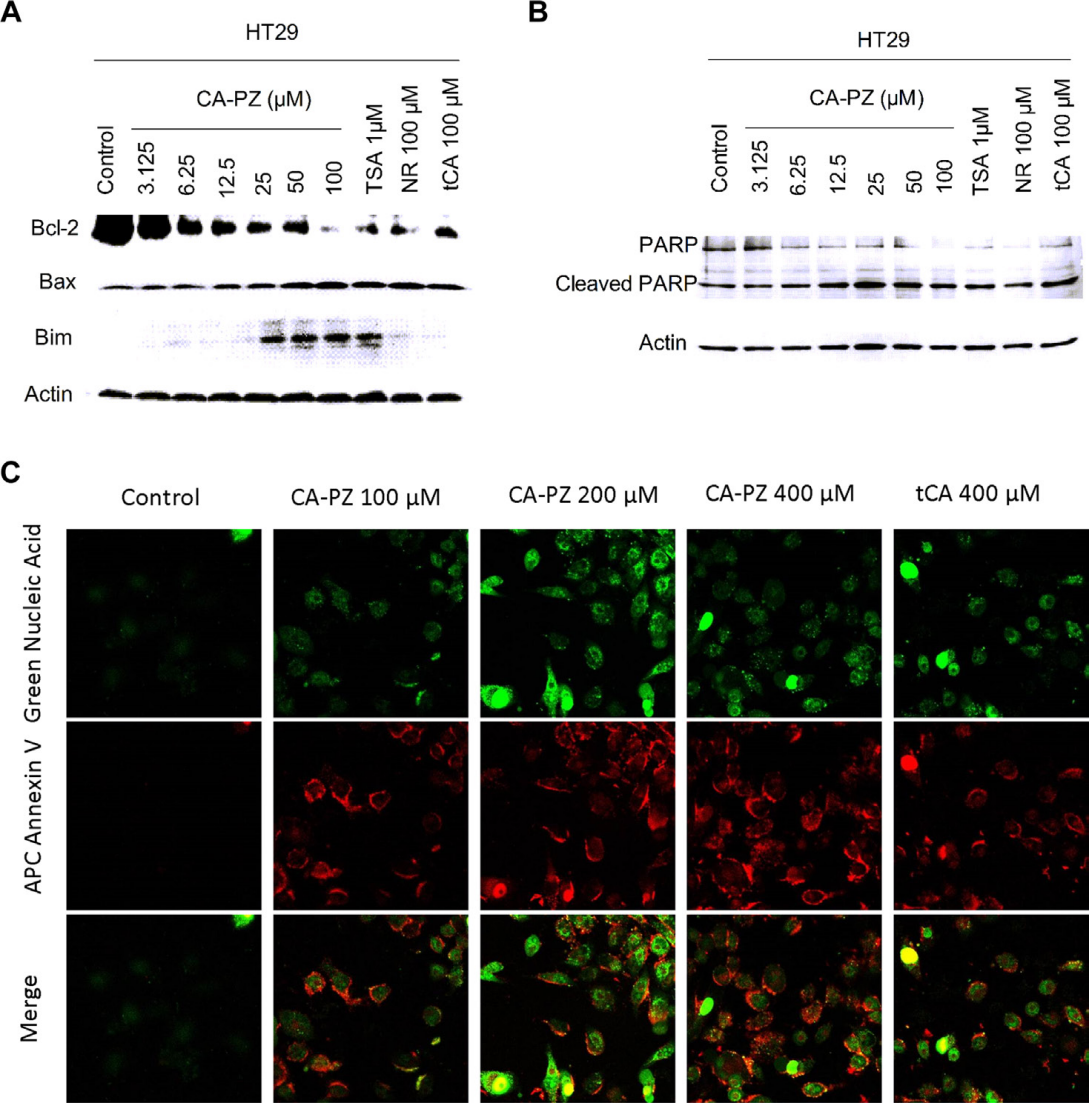
Western blot analysis on the expression levels of apoptosis-related proteins and the staining of apoptotic cells (**A**) CA-PZ at a wide range of concentrations for 12 h caused a marked increase in the expression levels of Bax and Bim, while a decrease in the expression level of Bcl-2 in HT29 cells. As shown, tCA at 100 μM, NR at 100 μM, and the known HDAC inhibitor TSA at 1 μM respectively caused changes similar to that of CA-PZ. (**B**) A decrease of PARP and an increase of cleaved PARP in HT29 cells were found after exposure to different concentrations of CA-PZ, the changes were similar to those induced by the positive control TSA. (**C**) MIA PaCa-2 cells treated with various concentrations of CA-PZ (100 μM, 200 μM, and 400 μM) for 12 h underwent apoptosis as determined with APC Annexin V and SYTOX^®^ green nucleic acid staining (original magnification 630×). Phosphatidylserine (PS) which translocated to the external leaflet in CA-PZ treated cells was stained by Annexin V, indicating apoptotic cells.

The induction of apoptosis by CA-PZ in HT29 cells and MIA PaCa-2 cells was determined. In HT29 cells, CA-PZ caused a significant increase in the expression levels of Bax and Bim, while a significant decrease in the expression level of Bcl-2. Similar changes were found in the cells treated by tCA at 100 μM, NR at 100 μM, and the known HDAC inhibitor TSA at 1 μM, respectively. In addition, a decrease of PARP and an increase of cleaved PARP occurred in HT29 cells after exposure to CA-PZ, these changes were similar to those caused by the positive control TSA (Figure 5A and 5B). The cells undergoing apoptosis were observed by the APC Annexin V staining. A significant increase of APC Annexin V stained cells was detected in MIA PaCa2 cells after CA-PZ treatment (Figure 5C).

### Tumor localization and accumulation

An optical molecular imaging system was used to evaluate the tumor localization and CA-PZ accumulation in colon carcinoma HT29 and pancreatic carcinoma MIA PaCa-2 xenograft-bearing mice. As shown (Figure 2C), CA-PZ penetrated into in the HT29 and MIA PaCa-2 tumor xenografts within 1 hour after i.v. injection and then most of the fluorescence signal of CA-PZ gradually localized and accumulated in the tumors. CA-PZ accumulation reached the highest level within 2–3 h after injection and then faded within 8 h. This observation confirmed the specific distribution of CA-PZ, a small molecule compound, in colon carcinoma HT29 and pancreatic carcinoma MIA PaCa-2 xenografts.

### Therapeutic efficacy of CA-PZ against colon carcinoma HT29 and pancreatic carcinoma MIA PaCa-2 xenografts

**Figure 6:**
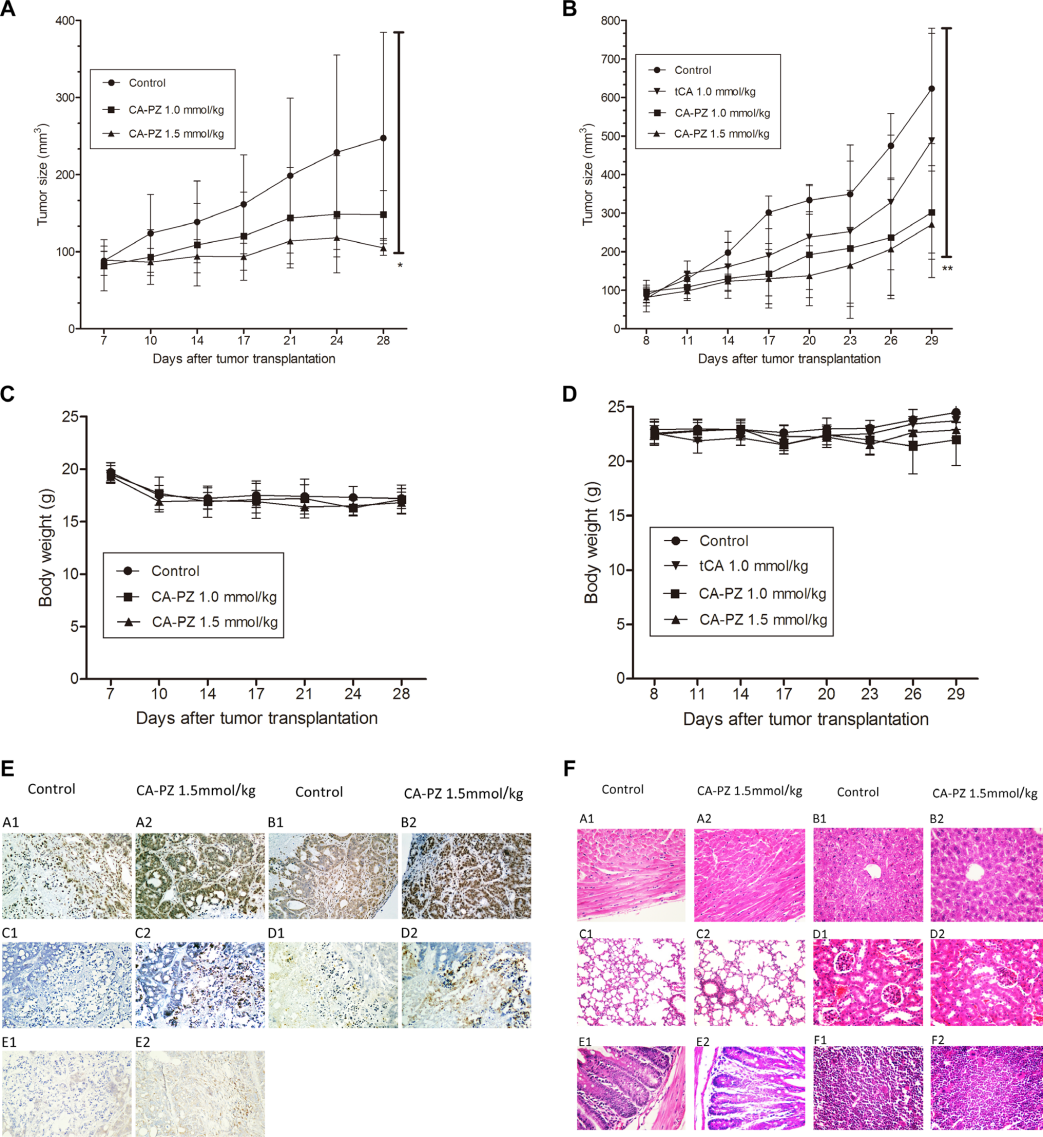
Anti-tumor efficacy of CA-PZ against human colon carcinoma HT29 and MIA PaCa-2 pancreatic carcinoma xenograft in athymic mice (**A**) and (**B**) The growth curves of MIA PaCa-2 and HT29 xenografts in different groups (*n* = 6) respectively are shown. Tumor volumes are measured every 3 days after treatment. As shown, CA-PZ at the tested doses suppressed the growth of tumors. Significant differences were found between the control group and the CA-PZ treated groups (*P < 0.05; **P < 0.01). (**C**) and (**D**) Body weight curves for the treated and the control animals respectively for MIA PaCa-2 and HT29 xenograft-bearing athymic mice. (**E**) Immunohistochemical staining of the HT29 carcinoma xenograft in CA-PZ treated group (1.5 mmol/kg) and the control group. (magnification 200×): A1 and A2, the IHC staining for Ac-H3; B1 and B2, the IHC staining for Ac-H4; C1 and C2, the IHC staining for cleaved PARP; D1 and D2, the IHC staining for cleaved caspase 3; E1 and E2, the IHC staining for Bim. (**F**) Histopathological appearance of various organs of the CA-PZ (1.5 mmol/kg) treated and the control xenograft-bearing mice. (H&E. staining, magnification 200×): A1 and A2, heart; B1 and B2, liver; C1 and C2, lung; D1 and D2, kidney; E1 and E2, small intestine; F1 and F2, femur bone marrow. No toxicopathological changes were found in all of the tested organs.

Pancreatic carcinoma MIA PaCa-2 and colon carcinoma HT29 xenograft models were used for evaluation of the therapeutic efficacy. As shown (Figure 6A and 6B), CA-PZ significantly suppressed the growth of pancreatic carcinoma MIA PaCa-2 and colon carcinoma HT29 xenografts. CA-PZ at doses of 1.0 mmol/kg and 1.5 mmol/kg inhibited tumor growth by 32.2% and 62.4% (*P* < 0.05) in pancreatic carcinoma MIA PaCa-2 xenograft, 52.6% (*P* < 0.05) and 58.8% (*P* < 0.01) in colon carcinoma HT29 xenograft, respectively; while tCA at 1.0 mmol/kg by 37.1% in colon carcinoma HT29 xenograft. During the experiment, no differences were found in daily food intake between the treated athymic mice and the controls. No deaths were found in all groups of treated and control mice. Moreover, no significant body weight changes occurred in the treated groups throughout the duration of the study (Figure 6C and 6D).

### Immunohistochemical examination on HT29 tumor tissue

To determine the changes of related proteins, immunohistochemical analysis was performed using specimens of HT29 colon carcinoma xenograft from the control and CA-PZ-treated athymic mice. Both Ac-H3 and Ac-H4 were overexpressed in CA-PZ treated HT29 carcinoma. Moreover, cleaved caspase 3 and cleaved PARP were overexpressed in CA-PZ treated HT29 carcinoma as compared with the control. In addition, the expression of Bim slightly increased after CA-PZ treatment (Figure 6E).

### Histopathological examination of various organs from the CA-PZ treated mice

At the end of experiment, specimens of various organs including the heart, liver, lung, kidney, small intestine, and femur bone marrow were taken from the CA-PZ treated (1.5 mmol/kg) mice and the controls. Histological sections of 5 μm in thickness were stained with H&E. and observed with microscope. No toxicopathological changes were found in the above-mentioned organs of CA-PZ treated animals (Figure 6F). The results suggested that the administrated doses of CA-PZ were well tolerated.

## DISCUSSION

The present study indicates that CA-PZ is active as HDAC inhibitor in association with intensive macropinocytosis-mediated entry into cancer cells. By *in vivo* imaging, CA-PZ displayed prominent tumor localization and accumulation. CA-PZ exerted therapeutic efficacy against the pancreatic carcinoma MIA PaCa-2 and colon carcinoma HT29 xenografts in nude mice. Moreover, CA-PZ markedly decreased the protein expression levels of acetyl-H3, acetyl-H4 and p21, which was consistent with effects of the known HDAC inhibitor TSA, indicating that CA-PZ is an active HDAC inhibitor. Evidently, the newly synthesized compound CA-PZ retains the attributes of both NR and tCA, displaying enhanced macropinocytosis-mediated uptake by the cell and the effects of HDAC inhibitor. This bi-functional property of the chimeric molecule is important for the development of new cancer therapeutics, especially for the pancreatic ductal adenocarcinoma (PDAC), the most common type of pancreatic cancer. On one hand, macropinocytosis-mediated drug delivery may be used for targeting K-Ras related cancers. As reported, K-Ras mutation occurs in over 90% of PDAC. In this occasion, the oncogenic K-Ras stimulates macropinocytosis to support the nutrient need for cancer cell metabolism and proliferation. Enhanced macropinocytosis was found in K-Ras mutant pancreatic carcinoma cells; consequently, the intensity of macropinocytosis in K-Ras mutant carcinoma cells was much higher than that in wild type carcinoma cells [3]. Therefore, intensive macropinocytosis might be exploited to deliver massive payload of drugs to target cancer cells. In our previous study, a recombinant fusion protein that consists of β-defensin and albumin (DF-HSA) was prepared. As shown, DF-HSA massively enters into the K-Ras mutant pancreatic carcinoma cells. A specific distribution and accumulation in tumor xenograft was found by imaging. DF-HSA exerted potent therapeutic efficacy on the tumor xenograft [38]. On the other hand, there is evidence that HDAC inhibitors may play an active role in the treatment of pancreatic cancer. An investigation found that an HDAC inhibitor can prevent the smoking- induced promotion of pancreatic carcinoma [39]. An HDAC inhibitor was reported to be effective in a PDAC mouse model [40]. As shown, targeting K-Ras signaling alone has limited effects against PDAC in mouse models; however, targeting HDAC in combination with K-Ras targeting markedly enhanced the efficacy [41]. Above-mentioned studies highlight the relevance of macropinocytosis to K-Ras mutant pancreatic carcinoma and the great potential of HDAC inhibitors in treatment of this refractory cancer. Therefore, the chimeric compound CA-PZ warrants further evaluation.

Neutral red (NR) is a well-known agent used in cytotoxicity assay for drug screening on the basis that NR can be internalized into living cells. The intracellular entry of NR can be blocked by the inhibitor EIPA; apparently, this is a macropinocytosis-mediated endocytic process. In comparison with other endocytic routes, macropinocytosis is unique for its selectivity and the large payload. In the present study, NR as a “guided module” was linked to an “effector module” for constitution of a bi-functional chimeric molecule. Macropinocytosis poses to be an efficient path for drug delivery. To our knowledge, this is the first report on the use of NR to prepare macropinocytosis-mediated HDAC inhibitor. The use of neutral red for preparation of macropinocytosis-mediated agents might open an alternative way for development of highly effective HDAC inhibitors and relevant targeted drugs.

## MATERIALS AND METHODS

### Reagents

The reagents and antibodies used in the study are as follows: fetal bovine serum (Gibco, USA), RPMI 1640 medium (Gibco; Life Technologies, CA, USA), Dulbecco's modified Eagle medium (Hyclone; Thermo Fisher Scientific, MA, USA), MTT (Amresco Inc, Solon, OH, USA), penicillin G and streptomycin (North China Pharmaceutical Co., Ltd, Shijiazhuang, China), polyvinylidene difluoride membranes (Millipore, Billerica, MA, USA), citrate buffer (Zhongshan Goldenbridge Biotechnology Co., Ltd., Beijing, China), and hematoxylin (Sigma-Aldrich, St. Louis, MO, USA). All the antibodies were purchased from Cell Signaling Technology, USA. The antibodies include Ac-H3, acetyl-histone H3 (Lys9) rabbit mAb 1:1000 (#9649, CST); Ac-H4, acetyl-histone H4 (Lys8) rabbit polyclonal antibody 1:1000 (#2594, CST); Actin rabbit polyclonal antibody 1:5000 (SC-1616, Santa Cruz); PARP polyclonal antibody 1:1000 (#9542, CST); Bax rabbit mAb 1:1000 (#5023,CST); Bcl-2 rabbit mAb 1:1000 (#2870, CST); and goat anti-rabbit peroxidase-coupled antibody (Zhongshan Golden Bridge Biotechnology, diluted 1:5,000). APC Annexin V was purchased from Invitrogen. Fluorescein isothiocyanate dextran and tCA were purchased from Sigma. 5-(N-ethyl-N-isopropyl) amiloride (EIPA) was from Life Technologies (Beijing).

### Synthesis and purification

The condensing reaction was performed between cinnamoyl chloride and neutral red (NR) which is the hydrochloride of 2-amino-8-dimethylamino-3-methylphenazine (PZ), in the alkaline solvents N, N-dimethylformamide (DMF) and triethylamine. The ratio of NR and cinnamoyl chloride was 1:2.2. By stirring at 58–61°C for about 72 h, the formed product CA-PZ was extracted with 30 ml benzene and 60 ml diethyl ether under stirring at room temperature. After filtration, the filtrate was put into a dryer at 60°C for 2–4 h. Then the crude product CA-PZ was purified through column chromatography with the solvent systems of EA:Bz:MeOH (3:2:0.5), EA:Bz:MeOH (3:1:0.6) and EA:Bz:MeOH (3:1:3), respectively.

### Macropinosome visualization

HT29 and MIA PaCa-2 cells were seeded onto the Thermo Scientific Nunc Lab-Tek II chamber slides. Twenty-four hours after seeding, CA-PZ, NR, tCA and EIPA were added respectively. After 2 h, the supernatant was discarded and cells were rinsed three times with cold PBS and then stained with DAPI; then rinsed three times in cold PBS, and immediately fixed for 15 min in 4% formaldehyde. Images were captured by confocal microscopy LSM 710 (Zeiss, Weimar, Germany). Because CA-PZ and NR have spontaneous fluorescence, macropinosomes could be seen without labeling agent.

### Cell culture

A number of human cancer cell lines have been used in the study. The cell lines include pancreatic carcinoma MIA PaCa-2 cells, colon carcinoma HT29 and HCT116 cells, lung carcinoma A549 and H460 cells, epidermoid carcinoma A431 cells, ovary carcinoma OVCAR-3 cells, and fibrosarcoma HT1080 cells. In addition, the non-cancerous hepatocyte L02 cells were used. All the cell lines were provided by the Cell Center of the Institute of Basic Medical Sciences, Chinese Academy of Medical Sciences and Peking Union Medical College. HCT116 cells, HT29 cells, A431 cells, A549 cells, H460 cells, OVCAR-3 cells, HT1080 cells and L02 cells were grown in modified RPMI-1640 supplemented with 10% heat-inactivated fetal bovine serum, penicillin G (100 U/mL), and streptomycin (100 μg/mL). MIA PaCa-2 cells were grown in Dulbecco's modified Eagle medium supplemented with the same substances as above-mentioned. All cell lines were cultured at 37°C in a humidified 5% CO_2_ incubator.

### MTT assay

Cells were plated in 96-well plates at a density of 3000–5000 cells per well and incubated at 37°C for 24 h. Then different concentrations of CA-PZ were added respectively which was dissolved by DMSO in the ratio of 1:1000. At 48 h, MTT was added to each well and further incubated for 4 h. Then, the medium containing MTT solution was discarded, and 150 μL of DMSO were added to each well. The absorbance (A) at 570 nm was detected with a microplate reader (Multiskan MK3; Thermo Fisher Scientific, MA, USA). Untreated cells were served as the control. The CA-PZ relative cell viability (%) compared with the control group was calculated according to the formula: cell viability (%) = [(A_treat_− A_blank_)/(A_control_ − A_blank_)] × 100%.

### Western blot analysis

After incubation at 37°C for 12 h, ice-cold PBS solution was used to rinse the cells three times. Then cells were lysed with the lysis buffer and the dishes were incubated for 5–10 min at 4°C. The cells were scraped into lysis buffer, and the lysates were clarified by centrifugation (15, 294 × g, 10 min at 4°C). Protein concentrations were determined using a BCA Protein Assay Kit from Bio-Rad and Western blot was performed. Briefly, an equal amount of total protein extracted from cultured cells was separated by 12% SDS-PAGE and transferred to the polyvinylidene difluoride (PVDF) membranes. Primary antibodies and horseradish peroxidase (HRP)-conjugated appropriate secondary antibodies were used to detect the designated proteins. After shaking slightly at 4°C overnight, the secondary antibodies bound on the PVDF membrane were reacted to the ECL detection reagents and exposed to X-ray films (Kodak, Japan). Antibody bound proteins were detected by enhanced chemiluminescence reagents (Pierce).

### Cell apoptosis assay

This was performed using APC Annexin V and SYTOX^®^ Green Nucleic Acid staining. In brief, cultured HT29 cells were washed with cold PBS and the binding buffer was added, then incubated with APC Annexin V for 10 min and stained with SYTOX^®^ Green Nucleic Acid Stain for another 10 min at 4°C in dark. Then, the stained cells were observed and photographed with confocal microscopy within 60 min.

### Localization of CA-PZ by *in vivo* optical fluorescence imaging

Tumor localization of CA-PZ was investigated using HT29 and MIA PaCa-2 xenografts in athymic mice. Cells were inoculated to the right armpit of athymic mice. When the solid tumors reached a volume of about 100–400 mm^3^, CA-PZ which is with spontaneous fluorescence was injected into the tail veins of mice (*n* = 3) at a dosage of 1.5 mmol/kg. Next, the mice were anesthetized by isofluorane at several selected time points (1, 2, 3, 4, 6, and 8 h) and placed in the imaging chamber of an IVIS-200 system (Xenogen) for observation. Images and measurements of CA-PZ fluorescence signals were analyzed using Living Image-software (Xenogen) referred to *in vivo* imaging as described [38].

### Therapeutic experiment

Female BALB/c athymic mice (6–8 weeks old) were purchased from the Institute for Experimental Animals, Chinese Academy of Medical Sciences and Peking Union Medical College. For the sake of making mice adapted to the new environment, the mice were under observation for one week before the experiment. The experiment protocols were based on the Regulations of Good Laboratory Practice for Non-clinical Laboratory Studies of Drugs issued by the National Scientific and Technologic Committee of the People's Republic of China. Colon carcinoma HT29 cells or pancreatic carcinoma MIA PaCa-2 cells (1 × 10^7^) suspended in 200 μL sterile saline were inoculated s.c. in the right armpit of athymic mouse. About 3–4 weeks later tumors in the donor animals were aseptically dissected. After cautiously removing the necrotic portion, the tumor mass was cut into pieces of 2 mm^3^ in size. Then the tumor tissue piece was transplanted subcutaneously by a trocar needle into the athymic mice. When the tumor size reached approximately 100 mm^3^, mice were divided into several groups (*n* = 6), including those treated with different doses of CA-PZ (1.0 mmol/kg and 1.5 mmol/kg, respectively), tCA (1.0 mmol/kg) and the control group, which were dissolved by 0.5% sodium carboxymethylcellulose (CMC). The tested drugs were given by intragastric administration, 0.2 ml/20g, 3 times a week, for a total of 6 doses. The short and long diameters of HT-29 and MIA PaCa-2 tumor xenografts were measured every three days and the tumor volumes were calculated. Tumor volume was estimated by the following formula: V = 0.5*a* × *b*^2^, where *a* and *b* represent the long and the perpendicular short diameters of the tumor, respectively. Thirty-two days after tumor inoculation, the animals were euthanized by isoflurane anesthesia. The xenografts and specimens from various organs were preserved in a 4% formaldehyde solution for further examination.

### Immunohistochemistry

Specimens taken from the HT29 xenografts were used for immunohistochemical detection of various related proteins, such as acetyl H3, Bim, cleaved PARP and cleaved caspase 3. Sections of 4 μm in thickness were deparaffinized and rehydrated with xylene and graded alcohol solutions. After washing with PBS, endogenous peroxidase activity was quenched by 3% hydrogen peroxide, and the sections were boiled in 10 mM citrate buffer (pH 6.0) for 3–5 min in an autoclave sterilizer followed by cooling at room temperature for more than 30 min. After rinsing with PBS, the sections were incubated with primary antibodies (1:100 diluted in antibody diluent, Zhongshan Goldbridge Biotechnology Co., Ltd, Beijing, China) overnight at 4°C. Sections were stained with related antibodies, respectively. After rinsing with PBS, the sections were incubated with PV6001 or PV6002 (Zhongshan Goldbridge Biotechnology CO., Ltd, Beijing, China) for 1 h at 37°C and stained with DAB (AR1022, Boster Biological Technology, Ltd, Wuhan, China) for 1 to 2 min. The slides were counterstained with hematoxylin, dehydrated with graded ethanol, cleared with xylene, and mounted in neutral gum. All slides were analyzed by two independent observers.

### Data analysis

All values were expressed as mean ± SD. Statistical analysis is carried out by one-way ANOVA using the SPSS statistical software (SPSS17.0 Inc., Chicago, IL, USA). Probability values (*P*-value) < 0.05 is considered as statistically significant.
